# Exploring the Preparation of Albendazole-Loaded Chitosan-Tripolyphosphate Nanoparticles

**DOI:** 10.3390/ma8020486

**Published:** 2015-02-05

**Authors:** Bong-Seok Kang, Sang-Eun Lee, Choon Lian Ng, Jin-Ki Kim, Jeong-Sook Park

**Affiliations:** 1 College of Pharmacy and Institute of Drug Research and Development, Chungnam National University, 99 Daehak-ro, Yuseong-gu, Daejeon 305-764, Korea; E-Mails: happy.kbs86@gmail.com (B.-S.K.); nnininn@hanmail.net (S.-E.L.); janice180606@gmail.com (C.L.N.); 2 College of Pharmacy and Institute of Pharmaceutical Science and Technology, Hanyang University, 55 Hanyangdaehak-ro, Sangnok-gu, Ansan 426-791, Korea

**Keywords:** albendazole, chitosan, tripolyphosphate, nanoparticle, anti-cancer effect

## Abstract

The objective of this study was to improve the solubility of albendazole and optimize the preparation of an oral nanoparticle formulation, using β-cyclodextrin (βCD) and chitosan-tripolyphosphate (TPP) nanoparticles. The solubility of albendazole in buffers, surfactants, and various concentrations of acetic acid solution was investigated. To determine drug loading, the cytotoxic effects of the albendazole concentration in human hepatocellular carcinoma cells (HepG2) were investigated. The formulations were prepared by mixing the drug solution in Tween 20 with the chitosan solution. TPP solution was added dropwise with sonication to produce a nanoparticle through ionic crosslinking. Then the particle size, polydispersity index, and zeta potential of the nanoparticles were investigated to obtain an optimal composition. The solubility of albendazole was greater in pH 2 buffer, Tween 20, and βCD depending on the concentration of acetic acid. Drug loading was determined as 100 µg/mL based on the results of cell viability. The optimized ratio of Tween 20, chitosan/hydroxypropyl βCD, and TPP was 2:5:1, which resulted in smaller particle size and proper zeta positive values of the zeta potential. The chitosan-TPP nanoparticles increased the drug solubility and had a small particle size with homogeneity in formulating albendazole as a potential anticancer agent.

## 1. Introduction

Albendazole is a poorly water-soluble but highly permeable anthelmintic drug [1], classified as a type II drug based on the Biopharmaceutical Classification System. Recently, the anticancer effects of albendazole were investigated [2,3], however, its antitumor efficacy is limited by low solubility. Thus, an appropriate solubilization formulation of albendazole is necessary to increase drug efficacy.

Moreover, β-cyclodextrin (βCD) is an efficient carrier for the delivery of albendazole [4,5]. Cyclodextrins (CDs) are cup-shaped molecules with hydrophobic cavities and hydrophilic exteriors that can interact with various hydrophobic guest molecules to form supramolecular inclusion complexes [6,7]. CDs have been exploited to enhance the bioavailability of insoluble drugs by increasing drug solubility and permeability. Moreover, the safety of CDs in humans has been well established [6,8].

Various formulations such as liposomes [9], suspensions [10,11], solid dispersions [12], microspheres [13], and nanoparticles [14] have recently been developed for albendazole. Of these, chitosan-based drug delivery systems are of great interest due to their biocompatibility, prolonged drug release, and lack of toxicity. Chitosan is a biodegradable and biocompatible cationic polymer widely studied in the preparation of nanoparticles for drug delivery [15,16]. Chitosan is frequently used in the development of controlled drug delivery systems [17] due to its adhesive properties and the ability to enhance the penetration of large molecules across mucosal surfaces, demonstrating sustained release [18].

Nanoparticles can be prepared by electrostatic interaction and resultant ionotropic gelation between chitosan and the tripolyphosphate (TPP) polyanion [19,20]. This interaction requires only mild temperature and pH conditions [21] and the nanoparticle size can be controlled by varying the chitosan to TPP ratio, pH, and the molar mass of the chitosan [22]. Due to their submicron size, TPP-chitosan nanoparticles can penetrate into tissues via capillaries [23].

However, common methods such as TPP crosslinking, emulsification-solvent volatilization, and the macromolecular condensed method cannot be used to produce albendazole-associated chitosan nanoparticles because of the incompatibility of the drug and the carrier. Albendazole, which is relatively insoluble in water and most organic solvents, can be dissolved in an acid medium, such as glacial acetic acid. In addition, the chitosan carrier is largely inhibited by its solubility in weak acidic environments (pH < 6.0).

In the present study, we improved the solubility of albendazole using different surfactants and different pH conditions and prepared albendazole-loaded nanoparticles with adequate size distribution. The manufacturing parameters were optimized by determining compositions such as Tween 20, hydroxypropyl-β-cyclodextrin (HPβCD), chitosan, and TPP. Then, optimized formulation ratios were obtained based on the size and polydispersity index (PDI) of albendazole-loaded nanoparticles.

## 2. Materials and Methods

### 2.1. Materials

Albendazole was a gift from Kolmar Korea (Sejong, Korea). Chitosan (MW = 140 kDa, 85% deacetylated), sodium tripolyphosphate (TPP), Tween 20, hydroxyl-β-cyclodextrin (HPβCD), (2,6-di-*O*-methyl)-β-cyclodextrin (DMβCD), and 3-(4,5-dimethylthiazol-2-yl)-2,5-diphenyltetrazolium bromide (MTT) were purchased from Sigma-Aldrich (St. Louis, MO, USA). Dulbecco’s modified Eagle’s medium (DMEM), fetal bovine serum (FBS), penicillin-streptomycin, and trypsin-EDTA were purchased from Gibco BRL (Grand Island, NY, USA). Distilled and deionized water was used after sterilization. All other chemicals were of reagent grade and used without further purification.

### 2.2. Cell Culture

Human hepatocellular carcinoma cells (HepG2) were cultured in DMEM supplemented with 10% heat-inactivated FBS, 100 U/mL penicillin and 100 μg/mL streptomycin at 37 °C in a humidified incubator with 5% CO_2_.

### 2.3. Solubilization of Albendazole in Buffers, Surfactants, and Various Concentrations of Acetic Acid Solution

An excess amount of albendazole was mixed with different pH buffers, surfactants, and concentrations of acetic acid in water by vortexing and was maintained at an ambient temperature for 3 days. For the solubility of albendazole in surfactants, the surfactant solutions were prepared 1% (w/v) in distilled water. To investigate the solubility of albendazole in acetic acid solutions, the concentration of acetic acid was varied from 1% to 50%. The equilibrated samples were centrifuged at 1000 rpm for 10 min to remove undissolved albendazole. The supernatant was filtered through a 0.45 µm PVDF syringe filter and the concentration of albendazole was determined using a spectrophotometer at 295 nm [24].

### 2.4. Cytotoxicity

Concentration-dependent cytotoxicity of albendazole was determined in HepG2 cells to verify the anticancer effects and loading amount of albendazole in nanoparticles. Cells were transferred from a 100-mm cell culture plate into a 96-well plate at a density of 1 × 10^4^ per well. After overnight incubation at 37 °C, the cells were exposed to different concentrations of 100 μL albendazole solubilized in 1% dimethylsulfoxide in media for 24 h. Then, the medium was removed and 100 μL MTT-containing medium (5 mg/mL) was added to the wells. Following 4 h incubation at 37 °C, the MTT-containing medium was carefully aspirated to avoid disturbing any formed formazan crystals and 100 μL MTT solubilization solution was added to each well. Plates were incubated at room temperature for 30 min and optical densities were determined at 570 nm using a microplate reader (Sunrise, Tecan Trading, Männedorf, Switzerland). Cell viability was expressed as a percentage of the untreated control cells.

### 2.5. Preparation of Albendazole-Loaded Chitosan-TPP Nanoparticles

Chitosan-TPP nanoparticles were prepared using a modified ionic gelation method [25]. Briefly, chitosan was dissolved at 0.2% (w/v) in 0.1 M acetic acid at pH 2.86 [23,26] and HPβCD was dissolved in the same solution. The solution was magnetically stirred for 3 h and then filtered to discard any undissolved chitosan. TPP was dissolved at 0.12% (w/v) in 0.1 M NaOH. Next, the TPP solution was added dropwise to 1 mL chitosan solution [27] and the resultant solution was incubated for 60 min. The final suspension was filtered. The reaction was performed at three different ratios to evaluate the effects of the TPP-to-chitosan ratio on nanoparticle size and polydispersity index. Drug-loaded nanoparticles were prepared by solubilizing albendazole at 0.5% (w/v) in 2% Tween 20. After the various ratios of albendazole solution were added to the chitosan solution under sonication for 30 min at 25 °C, the TPP solution was mixed with the chitosan solution and albendazole under sonication for 30 min at 40 °C. Finally, nanoparticles were purified by centrifugation for 30 min at 15,000 rpm and then washed twice with distilled water. The resulting particles were lyophilized.

### 2.6. Measurement of Particle Size and Polydispersity Index

The particle size distribution and polydispersity index of nanoparticles were determined using a light-scattering spectrophotometer (Zetasizer Nano S90, Malvern Instruments Ltd., Malvern, UK). The zeta potential of nanoparticles was measured using a light-scattering spectrophotometer (ELS-Z, Otsuka, Japan). The samples were diluted with deionized water and then transferred in a quartz cuvette in the light scattering instrument to measure particle size and zeta potential, respectively. The stability of chitosan-TPP nanoparticles containing albendazole was investigated for 4 weeks by measuring particle size and the polydispersity index.

### 2.7. Statistical Analyses

Statistical analyses were performed using Student’s *t*-test or one-way analysis of variance (ANOVA). A *p*-value of <0.05 was considered to indicate statistical significance. All data are expressed as means ± standard deviations (SDs) from three independent experiments.

## 3. Results and Discussion

### 3.1. Solubility of Albendazole

Incorporating the drug into the innermost phase of the nanoparticles produces the optimal benefits of nanoparticle formulations. Because this process depends on the solubility of the drug, the solubility of albendazole in various pH buffers and surfactants was evaluated as a first step for optimizing the nanoparticle formulation. The solubility of albendazole was greater in pH 2 buffer (23.5 µg/mL), which was 2.7–17.7 times higher than in the other buffers (Table 1). A minimum solubility of albendazole was found at pH 8 and solubility remained low over the pH 4–10 range, which was considered in the preparation of albendazole-loaded chitosan-TPP nanoparticles because chitosan is generally solubilized in 1% acetic acid. Solubility of ricobendazole, a metabolite of albendazole, showed a U-shaped pH solubility profile similar to albendazole, which suggests that these drugs possess both acidic and basic groups [28]. Then the acid pH was determined as a preparation condition of albendazole-loaded chitosan-TPP nanoparticles. Regarding surfactants, Tween 20 was more effective for enhancing the solubility of albendazole, at 42.0 µg/mL, than other surfactants, although Tween 80 and Cremophor showed high solubility concurring with Mukherjee and Plakogiannis [29], who found low solubility of albendazole in similar solubility enhancers. When albendazole was solubilized in 1% solution of α-, β-, or γ-cyclodextrin, the solubilizing ability of β-cyclodextrin was higher than other cyclodextrin derivatives (Table 1). We considered the structural dimension of β-cyclodextrin appropriate to form an inclusion complex with albendazole, resulting in improved solubility. To prepare chitosan-TPP nanoparticles, we determined the solubility of albendazole in acetic acid, generally used for solubilizing chitosan. When the concentration of acetic acid was increased from 1% to 50% (v/v), the solubility of albendazole was improved from 9.69 ± 1.06 to 3808.87 ± 112.05 µg/mL (Table 2). The solubility of albendazole was improved by mixing acetic acid and HPβCD up to 1970 ± 140 µg/mL [29]. Therefore, the optimized mixing of acetic acid and derivatives of β-cyclodextrin was hypothesized to improve the solubility of albendazole.

**Figure 1:**
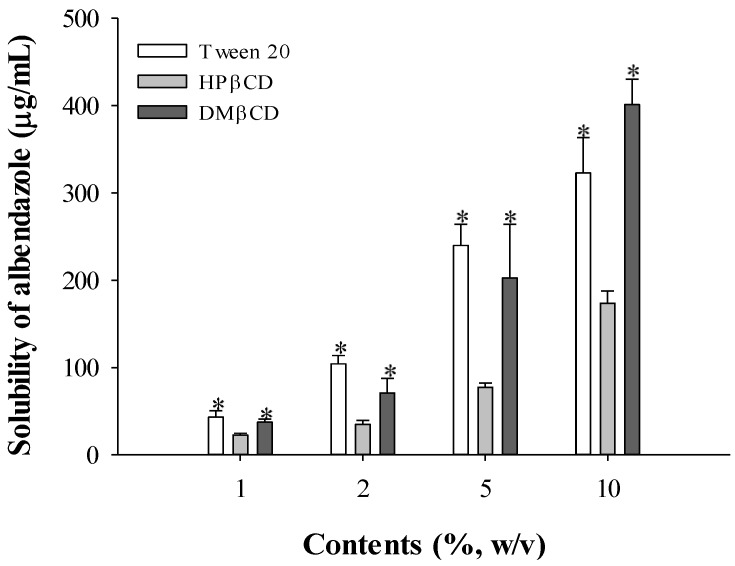
Effect of Tween 20 and cyclodextrins on the solubility of albendazole (*n* = 3). Note: * Significant difference compared to the treatment of Tween 20 in each content.

To maximize the solubilizing effect of Tween 20 and β-cyclodextrin, the effects of Tween 20, HPβCD, and DMβCD on the solubility of albendazole were investigated by varying the concentration (Figure 1). When increasing concentrations of Tween 20, HPβCD, and DMβCD from 1% to 10%, the solubility of albendazole was improved from 43.0 ± 7.3, 22.6 ± 2.0, and 37.5 ± 3.2 to 322.9 ± 40.6, 173.7 ± 14.0, and 401.3 ± 28.9 µg/mL, respectively. Based on the results shown in Figure 1, Tween 20 and HPβCD were selected for further study as a surfactant and solubilizing complex, respectively. Although the solubilizing capacity of DMβCD was higher than HPβCD, HPβCD was chosen after considering the manufacturing cost and the enhanced solubilizing effects when mixed with other additives.

**Table 1:** Effects of different pH and surfactants on the solubility of albendazole (*n* = 3).

Formulation		Solubility (μg/mL)
Buffer pH	pH 2	23.54 ± 2.94
	pH 4	8.63 ± 0.64
	pH 6	8.04 ± 0.26
	pH 8	1.33 ± 0.13
	pH 10	3.70 ± 0.05
Surfactant *	Tween 20	42.03 ± 9.49
	Tween 60	8.97 ± 1.70
	Tween 80	23.58 ± 0.94
	Span 80	4.50 ± 1.11
	Arlacel 80	14.07 ± 3.50
	α-cyclodextrin	3.30 ± 0.07
	β-cyclodextrin	12.25 ± 0.22
	γ-cyclodextrin	1.33 ± 0.00
	Poloxamer 188	2.83 ± 0.44
	Poloxamer 407	2.99 ± 0.75
	Cremophor RH 40	24.48 ± 8.07
	Eudragit L-100	2.48 ± 0.44

Note: * Concentration of surfactants was applied as 1% (v/v or w/v).

**Table 2:** Solubility of albendazole in different concentrations of acetic acid (*n* = 3).

Concentration of Acetic Acid (%)	Solubility (μg/mL)
1	9.69 ± 1.06
2	9.80 ± 4.87
3	15.15 ± 0.85
4	16.66 ± 0.97
5	22.54 ± 0.37
6	26.83 ± 0.69
7	34.11 ± 0.34
8	38.05 ± 1.22
9	43.06 ± 1.66
10	52.01 ± 4.53
15	115.26 ± 6.79
20	215.98 ± 11.72
25	462.80 ± 26.24
30	755.18 ± 37.30
35	1139.70 ± 70.30
40	1657.34 ± 68.86
45	2784.98 ± 159.27
50	3808.87 ± 112.05

### 3.2. Dose-Dependent Anticancer Effects of Albendazole

**Figure 2:**
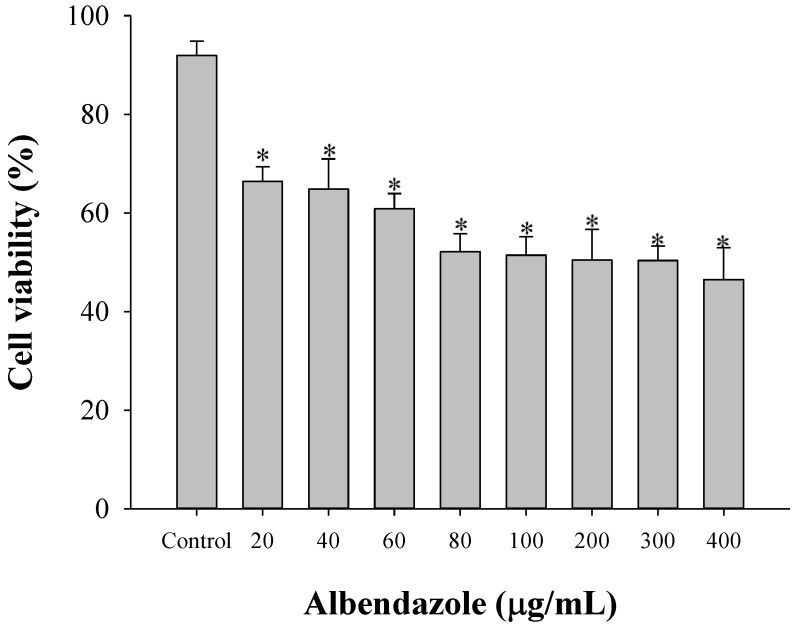
Cytotoxicity of albendazole at various concentrations (*n* = 5). Control means the group treated with 1% DMSO in culture media without albendazole. * Significant difference compared to the control group.

Cell viability in HepG2 was evaluated to determine the anticancer effects of albendazole (Figure 2). Cell viability of albendazole was 79.1% ± 5.1% when 100 µg albendazole per well was suspended in distilled water [30]. However, the HepG2 cell viability of albendazole was 66.4% ± 3.0% when 20 µg albendazole per well solubilized in 1% DMSO in culture media was added as shown in Figure 2. The anticancer effect of solubilized albendazole was considered more potent than the suspended drug. When the solubility of albendazole in 5% DMSO media was approximately 0.9 g/mL [31], it was confirmed that 400 µg/mL albendazole was completely solubilized. Therefore, the decreased cytotoxicity of albendazole was not due to the insolubility of the drug. To further evaluate the formulation, the loading amount of albendazole was fixed at 100 µg/mL.

### 3.3. Optimization of the Tween 20 to HPβCD Chitosan Ratio Based on the Solubility of Albendazole

**Figure 3:**
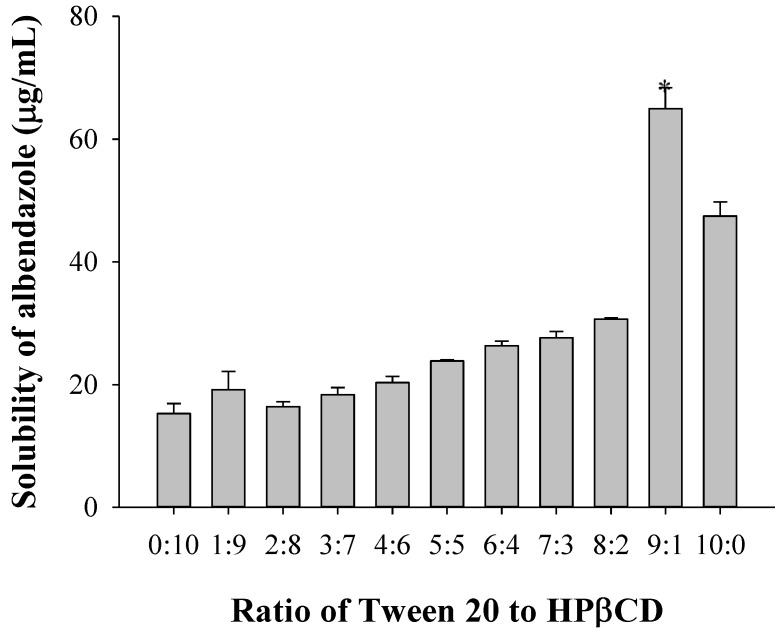
Solubility of albendazole depending on the Tween 20-to-hydroxyl-β-cyclodextrin (HPβCD) ratio (*n* = 3). Note: * Significant difference compared to all the other groups.

The formulation ratio of Tween 20 to HPβCD was optimized based on the solubility of albendazole; 1 g albendazole was solubilized in 2% Tween 20 or 1% HPβCD solution. To obtain an optimized Tween 20-to-HPβCD ratio, 2% Tween 20 and 1% HPβCD were mixed in various ratios from 1:9 to 9:1 for the preparation of the mixed solution for the solubility test of albendazole. As shown in Figure 3, the highest solubility of albendazole was obtained at a 9:1 ratio of Tween 20 to HPβCD. Therefore, the optimized Tween 20-to-HPβCD ratio was 9:1, which resulted in a higher solubility of albendazole.

### 3.4. Optimization of the Formulation Based on Particle Size and Polydispersity Index

**Figure 4:**
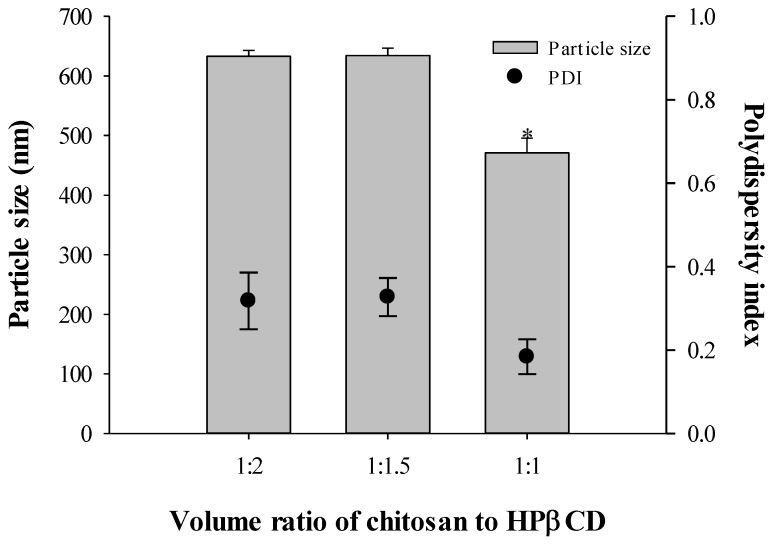
Effect of chitosan-to-HPβCD ratio on the size and polydispersity index of chitosan nanoparticles in various concentrations of loading TPP solution (*n* = 3). Note: * Significant difference compared to all the other groups.

The formulation ratio of chitosan to HPβCD was optimized based on the nanoparticle size and polydispersity index. Nanoparticles were prepared by varying the volume ratio of chitosan to HPβCD (1:2, 1:1.5, and 1:1) without drug loading. Concentrations of loading TPP solution were varied (1.2 mg/mL, 10 mg/mL, and 20 mg/mL). When 1.2 mg/mL TPP solution was loaded to the mixture of chitosan and drug, the nanoparticle size was 203.5 ± 3.53 nm. However, >10 mg/mL TPP solution did not produce particles due to its viscous properties. Therefore, the concentration of TPP applied was 0.12% (w/v). The particle sizes of drug-free nanoparticles were 633, 634, and 471 nm, and their polydispersity indices were 0.318, 0.327, and 0.184, respectively, based on the chitosan-to-HPβCD ratio (Figure 4). The optimized ratio of chitosan to HPβCD was 1:1, which resulted in the smaller particle size and lowest polydispersity index.

**Figure 5:**
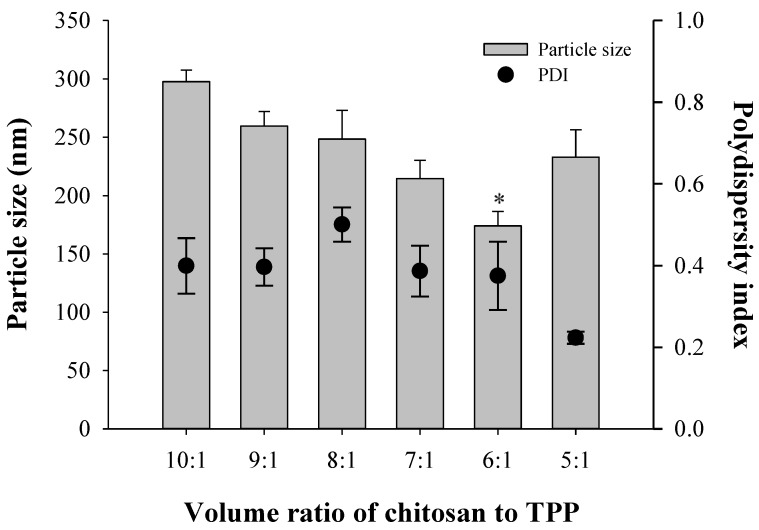
Effect of chitosan-to-TPP ratio on the size and zeta potential of chitosan nanoparticles (*n* = 3). Note: * Significant difference compared to all the other groups.

The ratio of chitosan to TPP was optimized based on the nanoparticle size and polydispersity index. Nanoparticles were prepared by varying the volume ratio of chitosan to TPP at 10:1, 9:1, 8:1, 7:1, 6:1, and 5:1 without drug loading. The particle sizes of drug-free nanoparticles were 298, 260, 248, 215, 174, and 233 nm, and their polydispersity indices were 0.399, 0.397, 0.501, 0.387, 0.375, and 0.223 based on the chitosan-to-TPP ratio (Figure 5). The optimized ratio of chitosan to TPP was 5:1, which did not result in a smaller particle size, but had the lowest polydispersity index.

**Figure 6:**
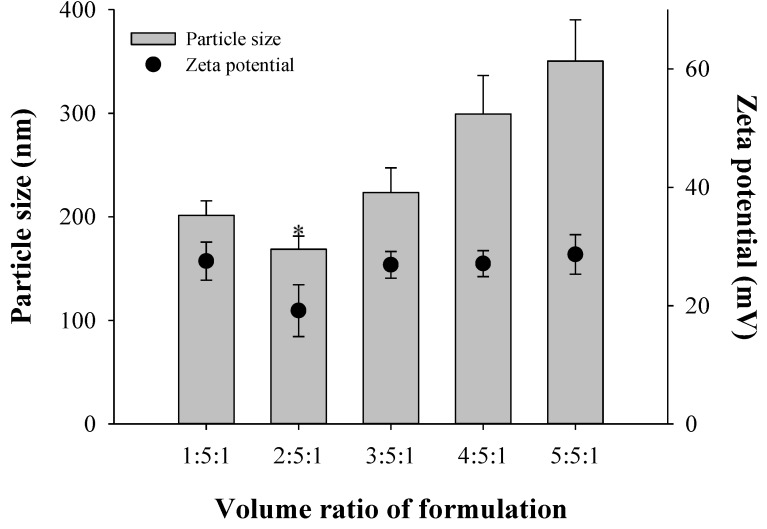
Effect of 2% Tween 20 solution including albendazole on the particle size and zeta potential (*n* = 3). The volume ratio of chitosan/HPβCD solution to chitosan-tripolyphosphate (TPP) solution was set as 5:1. Note: * Significant difference compared to all the other groups.

For complete optimization, the volume ratios of Tween 20, chitosan, and TPP were characterized based on the nanoparticle size and zeta potentials. Nanoparticles were prepared by varying the volume ratio of Tween 20, chitosan, and TPP (1:5:1, 2:5:1, 3:5:1, 4:5:1, and 5:5:1). Tween 20 solution contained 2% Tween 20 and 5 mg/mL albendazole. The sizes of drug-free nanoparticles were 201, 169, 224, 299, and 350 nm and their zeta potentials were 27.5, 19.1, 26.9, 27.1, and 28.6 based on the Tween 20, chitosan, and TPP ratios (Figure 6). The optimized ratio of Tween 20, chitosan, and TPP was 2:5:1, which resulted in a smaller particle size and appropriate positive zeta potential values.

### 3.5. Stability of Albendazole-Loaded Chitosan-TPP Nanoparticles

**Figure 7:**
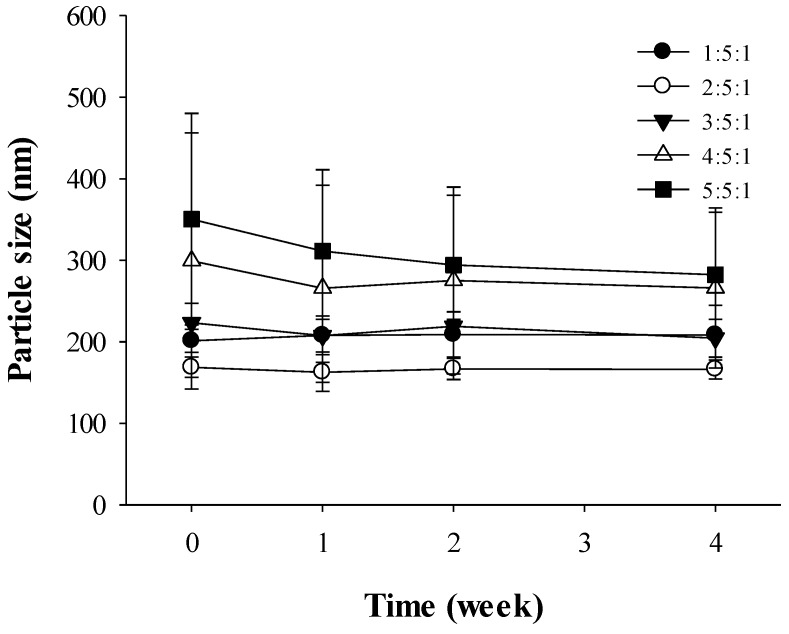
Stability of albendazole-loaded chitosan-TPP nanoparticles (*n* = 3). The volume ratio of chitosan/HPβCD solution to TPP solution was set as 5:1.

From the results in Figure 6, the formulation of chitosan-TPP nanoparticles was determined as 2:5:1 based on the smaller particle sizes of albendazole loaded nanoparticles. However, the stability of the nanoparticle formulations was studied to confirm the optimized formulation based on the changes in the particle sizes. Similar to the results from Figure 6, the particle sizes of drug-loaded nanoparticles were maintained consistently from 168.8 ± 12.6 nm at week 0 to 166.2 ± 11.5 nm at week 4 when the nanoparticles were prepared in the ratio 2:5:1 of Tween 20, chitosan, and TPP (Figure 7). However, large nanoparticles prepared at a 5:5:1 ratio decreased from 350.4 ± 129.9 nm at week 0 to 282.2 ± 76.8 nm at week 4. Although the nanoparticles prepared at the 5:5:1 ratio were larger at week 4 compared to those at the 2:5:1 ratio, the decreasing particle size pattern likely resulted from degradation due to low stability at that ratio.

## 4. Conclusions

To optimize the formulation of albendazole nanoparticles for oral delivery, we investigated the effective ratios of Tween 20, chitosan, and TPP. When the ratio of albendazole-loaded Tween 20 solution, chitosan HPβCD solution, and TPP solution was 2:5:1, a small particle size and low polydispersity index of nanoparticles was obtained. Moreover, the nanoparticles prepared using this formulation ratio were stable for four weeks. In conclusion, we determined the optimal composition of albendazole-loaded nanoparticles, which could be useful for the preparation of stable nanoparticles.
